# Within-Provider Variation in Prices for Commonly Utilized Services in Office Setting: Cross-Sectional Study

**DOI:** 10.2196/96883

**Published:** 2026-07-23

**Authors:** Yuvraj Pathak, David Muhlestein

**Affiliations:** 1 Simple Healthcare, 465 N Carolina Run, Sanford, FL, 32773, United States, 1 407-464-4444

**Keywords:** healthcare prices, price transparency, cost of care, transparency in coverage, healthcare price variation

## Abstract

Our study uses the latest Transparency in Coverage data to show that there is wide variation in health care prices for some of the most commonly utilized services in the United States. The findings demonstrate that there is an opportunity for employers, policymakers, and other stakeholders to curb health care spending by choosing cost-efficient health care networks and providers.

## Introduction

Health care prices in the United States have historically been hidden from the public hindering meaningful analysis of health care markets and prices. This matters because prices impact access to care and are a major driver of high health care spending [1,2].

The information landscape began to change with first price transparency rule, Hospital Price Transparency (HPT) in 2021 which required hospitals to disclose prices of a select set of services. Then, in 2022, the Transparency in Coverage (TiC) rule began to require insurers to release negotiated rates for all covered services.

These datasets have proven valuable in shedding light on variation in health care prices. HPT data has been used to demonstrate large variation in prices for urological procedures [3], and radiology services [4], among others. Recent studies have begun to exploit more comprehensive TiC data and show wide variation in prices for inpatient and outpatient services [5], within hospitals for commonly provided services [6], among others.

We assess the within-provider variation in prices for the top 20 most commonly services in an office setting and find that the average difference between the minimum and maximum rates is 40% of the national Medicare rate. Our results complement Wang et al (2024) study which examines within-hospital price variation for inpatient and outpatient services and finds that difference between minimum and maximum prices negotiated by hospitals to be 86% and 222% of Medicare rates, respectively [6].

## Methods

We downloaded the machine-readable files for the largest national payers (Cigna, Aetna, United Healthcare, various Blue Cross and Blue Shield plans) for the third quarter of 2025.

These files contain information on negotiated rates for all covered services along with identifying information on provider tax identification number (TIN), payer name and plan type.

We removed duplicates and ‘ghost rates’—negotiated rates corresponding to a provider for a service they are unlikely to ever bill for,—which are common in TiC data following the established methodology from published literature [7].

This analysis focuses on the top twenty most commonly utilized HCPCS ((Healthcare Common Procedure Coding System) codes in an office setting (determined based on national Medicare data and Colorado and New Hampshire’s All-Payer Claims databases). To limit the influence of outliers, we exclude rates below the 1st percentile and above the 99th percentile for each billing code and restrict the sample to billing code–TIN combinations with at least two negotiated rates.

The analysis data consists of approximately 5.6 million negotiated rates for 20 HCPCS codes in an office place of service. Table 1 shows the distribution of negotiated rates by billing codes.

**Table 1.** Percentiles and counts of negotiated rates by billing codes.

Billing code	Billing code description	Percentiles ($)					Standard deviation	Skewness	No. of negotiated rates
		10th	25th	50th	75th	90th			
36415	Collection of venous blood by venipuncture	$1.80	$2.91	$3.00	$5.50	$8.21	2.52	1.16	252,841
80048	Basic metabolic panel (BMP)	$4.23	$5.19	$6.77	$9.31	$12.82	3.56	1.44	129,614
80053	Comprehensive metabolic panel (CMP)	$5.08	$6.04	$8.12	$11.49	$15.65	4.46	1.42	136,811
80061	Lipid panel	$5.62	$7.74	$10.71	$15.00	$20.95	6.34	1.43	175,161
83036	Hemoglobin A1_c_ test	$4.67	$5.70	$7.77	$10.85	$14.86	4.24	1.42	183,042
84443	Thyroid stimulating hormone (TSH) test	$7.06	$9.65	$13.44	$18.28	$25.36	7.49	1.34	148,063
85025	Complete blood count (CBC) with differential	$4.63	$5.44	$6.29	$8.70	$11.96	3.15	1.47	187,763
85610	Prothrombin time (PT/INR)	$2.61	$3.00	$4.29	$5.50	$6.93	1.72	1.22	145,974
88305	Surgical pathology examination, moderate complexity (eg, biopsy tissue exam)	$40.94	$53.50	$71.84	$102.59	$131.49	41.50	1.43	42,117
90471	Immunization administration (1 vaccine)	$12.00	$14.72	$19.63	$26.80	$33.82	9.01	1.02	211,824
93010	Electrocardiogram (ECG/EKG) interpretation and report	$7.79	$9.36	$12.16	$14.94	$17.56	4.19	1.09	88,739
97110	Therapeutic exercises (physical therapy), 15 minutes	$20.80	$24.64	$29.66	$34.99	$45.91	10.72	1.61	215,203
97112	Neuromuscular reeducation, 15 minutes	$23.10	$27.31	$32.49	$38.96	$50.77	12.04	1.58	210,790
97140	Manual therapy techniques (eg, mobilization), 15 minutes	$19.14	$22.55	$27.24	$32.48	$42.59	10.20	1.62	218,521
97530	Therapeutic activities, 15 minutes	$24.93	$29.71	$36.50	$43.32	$56.53	13.60	1.46	145,810
98941	Chiropractic manipulation, 3‐4 spinal regions	$24.43	$30.65	$40.53	$54.19	$66.18	16.63	0.77	55,689
99203	New patient office visit, moderate complexity	$73.50	$86.70	$107.86	$130.09	$163.66	36.60	1.08	717,830
99212	Established patient office visit, low complexity	$28.66	$36.22	$46.53	$58.03	$72.91	17.42	0.89	708,342
99213	Established patient office visit, low-moderate complexity	$52.06	$61.28	$76.02	$94.90	$118.35	26.58	1.01	808,269
99214	Established patient office visit, moderate complexity	$75.95	$90.31	$110.02	$138.66	$172.76	38.73	1.03	805,806

We first calculate the mean, median, minimum, and maximum rates at the provider TINs and billing code level. These represent the calculated measures of prices a provider is willing to accept across insurers for a billing code.

We then take the average of these estimates across provider TINs at the billing code level to calculate the mean, median, minimum, and maximum rate a provider is willing to accept for a billing code on average.

We define the range of rates as the difference between the average minimum and average maximum rates divided by the national Medicare rate for each billing code.

### Ethical Considerations

This study is exempt from ethics review, as all data used is publicly available. The study uses data on health care prices released by insurers and does not involve any human subjects. It is thus exempt from institutional review board.

## Results

Table 2 shows the average mean, median, minimum, and maximum –negotiated rates for the top 20 billing codes. All four summary statistics are unweighted averages and represent the average of the respective summary statistics across all provider TINs at the billing code level. Out of the 20 billing codes, difference between the maximum and minimum rate is greater than or equal to 50% of the national Medicare rate for four billing codes - 80048 (Basic Metabolic Panel), 80061 (Lipid Panel), 85610 (Prothrombin Time), and 88305 (Surgical Pathology).

**Table 2.** Average of the mean, median, minimum, maximum values, and estimated range of negotiated rates, by billing code.

Billing code	Billing code description	Mean	Median	Minimum	Maximum	Medicare rate	% (Max. Min.) ÷ Medicare	Standard deviation	Skewness
		(averaged over provider TINs)^a^							
36415	Collection of venous blood byvenipuncture	$4.36	$4.29	$3.18	$5.64	$9.09	27	2.52	1.16
80048	Basic metabolic panel (BMP)	$7.97	$7.88	$5.94	$10.14	$8.46	50	3.56	1.44
80053	Comprehensive metabolic panel (CMP)	$9.30	$9.25	$7.15	$11.54	$10.56	42	4.46	1.42
80061	Lipid panel	$12.42	$12.20	$9.02	$16.15	$13.39	53	6.34	1.43
83036	Hemoglobin A1c test	$8.55	$8.48	$6.70	$10.49	$9.71	39	4.24	1.42
84443	Thyroid stimulating hormone (TSH) test	$14.97	$14.97	$11.21	$18.76	$16.80	45	7.49	1.34
85025	Complete blood count (CBC) with differential	$7.72	$7.60	$6.03	$9.61	$7.77	46	3.15	1.47
85610	Prothrombin time (PT/INR)	$4.61	$4.36	$3.41	$6.16	$4.29	64	1.72	1.22
88305	Surgical pathology examination, moderate complexity (eg, biopsy tissue exam)	$75.08	$74.35	$54.11	$97.22	$69.54	62	41.50	1.43
90471	Immunization administration (1 vaccine)	$19.42	$19.38	$15.04	$23.87	$20.05	44	9.01	1.02
93010	Electrocardiogram (ECG/EKG) interpretation and report	$10.67	$10.64	$9.27	$12.13	$7.76	37	4.19	1.09
97110	Therapeutic exercises (physical therapy), 15 minutes	$28.19	$28.05	$23.59	$33.00	$28.79	33	10.72	1.61
97112	Neuromuscular reeducation, 15 minutes	$31.37	$31.18	$26.36	$36.62	$32.02	32	12.04	1.58
97140	Manual therapy techniques (eg, mobilization), 15 minutes	$26.06	$25.90	$21.75	$30.57	$27.17	32	10.20	1.62
97530	Therapeutic activities, 15 minutes	$34.68	$34.43	$28.86	$40.79	$34.61	34	13.60	1.46
98941	Chiropractic manipulation, 3‐4 spinal regions	$39.45	$38.50	$30.83	$49.27	$38.49	48	16.63	0.77
99203	New patient office visit, moderate complexity	$116.01	$115.58	$99.79	$132.71	$109.01	30	36.60	1.08
99212	Established patient office visit, low complexity	$50.47	$50.37	$41.52	$59.46	$54.99	33	17.42	0.89
99213	Established patient office visit, ow-moderate complexity	$83.27	$83.02	$70.93	$95.93	$88.95	28	26.58	1.01
99214	Established patient office visit, moderate complexity	$121.03	$120.60	$103.78	$138.77	$125.18	28	38.73	1.03

a TIN: tax identification number.

Across the 20 billing codes, we find the average variation between the minimum and maximum rates to be 40 % of the Medicare rate (unweighted).

## Discussion

Our assessment shows that providers negotiate a wide range of prices for the same service across insurers. Thus, the same provider can be low cost for one network but high cost for another network. Our findings that prices for commonly utilized services can vary by up to 40% of the Medicare rate in an Office setting compliments existing literature that documents substantial variation in prices across and within hospitals. This highlights the importance of the choice of network and benefit design in reducing health care spending. For employers, health plans, and policymakers, our findings demonstrate that choice of networks and providers can have a substantial impact on health care spending. Future work should aim to understand how navigating patients to lower cost providers can generate potential savings.

## Abbreviations

HCPCS: Healthcare Common Procedure Coding System
HPT: Hospital Price Transparency
TiC: Transparency in Coverage
TIN: tax identification number
